# Editorial: Regulatory science and meta science as components of regulation of medical products and practices

**DOI:** 10.3389/fmed.2025.1664402

**Published:** 2025-08-12

**Authors:** Barbara K. Redman, Christine Gispen-de Wied, Thalia Arawi

**Affiliations:** ^1^Department of Population Health, Division of Medical Ethics, New York University Grossman School of Medicine, New York, NY, United States; ^2^Gispen4RegulatoryScience Consultancy, Bilthoven, Netherlands; ^3^T2P Center for Training and Consultancy, Beirut, Lebanon

**Keywords:** meta science, regulatory science, medical products, translating research, patient care practices

The inter-relationship between regulatory science and meta science shows great promise for accelerating promotion of high-quality research and the public's health. Defined narrowly by regulatory agencies as development of the tools and methods they use, regulatory science (RS) ought to more broadly describe responsibilities of regulatory agencies as well as self-regulation by professionals/scientists and citizen groups. It may therefore also reflect on its own performance and matters of improvement. It is thereby a moral framework and imperative to advance a distributed sense of responsibility and accountability as well as public trust in the regulatory ecosystem.

Regulatory goals require accurate, evidence-based understanding of the problem they are established to correct, not just, as has largely occurred, a politically motivated resolution or complete inattention. Meta-science (MS) is the study of science itself—a close cousin of evidence-based policy. It evaluates the quality of scientific practices including bias in the literature. Over time and especially recently, MS has uncovered long-standing flaws in the practice of science, which can undermine benefits the public should gain from its investment in research. In a way, it serves as the conscience of scientific practice, crucially ensuring that the foundations of medical regulation are rigorous and unbiased. Both RS and MS create value if competently practiced: both should be further developed individually and at their intersection.

The articles in this Research Topic are geographically diverse, each describing parts of their regulatory system that must be upgraded, at the level of country, region or continent, all enabled by the data revolution with most describing the need to harmonize and upgrade existing infrastructures. In other words, they are strengthening RS by using MS. An article from national regulatory authorities from 10 countries in West Africa aims to improve access to safe and effective medicinal products and health technologies for their populations by correcting critical regulatory gaps (Alfonso et al.). The East African Medicines Harmonization Initiative involves seven countries to allow joint reviews and inspections and to align with the African Medicines Agency (Ngum et al.). In both cases, the focus is on raising the maturity level of regulatory institutions, in part to address public health emergencies.

Other articles encompass policies for a continent or a union of countries (the EU), in recognition that an expanded and upgraded infrastructure is necessary. An article from Latin America offers a model that could be expanded to a global system for harmonization of minimum standards regulating biobanks, leading to higher-quality and more reliable data, including that needed for population health. Currently, each biobank in Latin America operates with its own set of standards, which makes reproducibility difficult. Common standards and capability are sorely needed to address urgent research needs (Valdes and Lecaros). Yet, a tailored approach to individual countries should remain in the realm of exploration as well. A one size fits all may not be the ultimate goal at the global level.

The European Innovative Health Initiative provides an example of a successful public-private partnership enabling access to large amounts of data meeting quality standards, necessary to speed innovation. Through a consortium agreement on data sharing and intellectual property rights, in a pre-competitive space, great progress can be made (Vaudano). Such public-private alliances are ethically beneficial for hastening useful innovations, if they operate under clear ethical guidelines for data privacy and access.

Two articles address deficiencies in tools necessary for the benefits of the RS-MS partnership to be harvested. Through MS, it has become clear that medical device regulations are complex and difficult to understand, affecting a wide range of stakeholders including manufacturers, regulators, health care professionals and patients, impeding their usefulness (Han et al.). Likewise, library and information science experts note how difficult it is to obtain full, complete literature reviews in the fields of RS and MS. Search terms and strategies are not optimized; multiple databases must be searched, each with their own search terms; gray literature is useful but difficult to obtain. Natural language processing and generative AI tools can be useful but must be further developed (Stevens and Laynor). Both of these areas must be upgraded to support appropriate evidence-based regulations.

It should also be noted that both RS and MS and the intersections between them, are often under-developed normatively. The paper on gaps in the ethical governance of pharmaceutical clinical trials in Europe notes partial alignment of European regulations with international ethical guidelines and limited post-approval oversight by RECs combined with insufficient connection with health inspectorates, and limited integration of ethical considerations in marketing authorization. These clearly identified gaps can undermine research participant safety and trust (Bernabe et al.). This article usefully draws attention to the need for development of norms to support expanded regulatory approaches.

And in a truly innovative approach, Perillat et al. suggest a new model to discuss whether individualized therapies should be considered and regulated as research or as treatment. This work offers a well-thought-out proposal to bridge these two worlds, for patient benefit and/or to produce generalizable knowledge to benefit future patients. It adds to ongoing work seeking clarification for both differentiating and harmonizing regulation of therapy and research (Perillat et al.).

Clearly, articles in this Research Topic, combining regulatory science with meta-science, have opened new horizons in at least three ways: (1) by clarifying needs for upgraded policies and regulatory infrastructures in all parts of the world, necessary to impact health goals, (2) by acknowledging how advances in MS and data science are essential to RS, and (3) by addressing empirical and normative shortcomings in current regulatory practices. Henceforth, many more examples should be analyzed and presented, leading to a more completely described landscape of synergies between RS and MS and capacity development in each.

While policy goals are defined politically, they will not be achieved unless policies are evidence-based (MS) and effectively embedded in regulation which is competently administered (RS). Ultimately, the robust integration of RS and MS, reinforced by a strong ethical framework, is, in addition to being a matter of scientific rigor and/or administrative efficiency, vital and necessary for safeguarding public trust, fostering justice, and ensuring that medical advancements really serve the public good.

